# The Intrauterine Bigatti Shaver System: An Alternative Option for Focal Retained Products of Conception

**DOI:** 10.1155/2018/1536801

**Published:** 2018-11-21

**Authors:** Neveta S. V. Sutherland, Hemashree Rajesh

**Affiliations:** ^1^Department of Obstetrics and Gynaecology, Victoria Jubilee Hospital, Kingston, Jamaica; ^2^Centre for Assisted Reproduction and Endocrinology, Singapore General Hospital, Outram Road, Singapore

## Abstract

The management of retained products of conception (RPOC) may be medical or surgical. Surgical options include blind curettage, ultrasound guided curettage, or curettage under direct vision via hysteroscopy. The definitive management of patients presenting with retained products of conception will depend on several factors: severity of bleeding, presence of hemodynamic instability or infection, and patient preference. Optimal management of retained products of conception should result in complete evacuation of the uterine cavity while minimizing endometrial trauma. This is of utmost importance in patients with reproductive desires. We report patients with RPOC managed via hysteroscopic removal using the Bigatti Morcellator. Both patients had complete evacuation of the visualized RPOC. The purpose of this paper is to present this approach as an effective management option particularly in patients with a history of subfertility and failed blind curettage.

## 1. Introduction

Retained products of conception (RPOC) refer to the presence of placental and/or fetal tissue remaining in the uterus after a gestational event [1]. Most commonly, patients present with abnormal uterine bleeding. The incidence varies and is dependent on several factors including the gestational age of presentation (more commonly in the second trimester and with terminated pregnancies), the initial mode of treatment (surgical versus nonsurgical), and duration of follow-up [1, 2]. Management may be expectant, medical, or surgical: each with its own merits and demerits [1–3]. Surgical management is traditionally via blind curettage and is considered the gold standard [3–5]. It is definitive and predictive, provides more rapid resolution of the pathology and a shorter convalescent period, and has a higher success rate compared to expectant or medical management [5]. Though traditional, blind curettage is not without risks such as incomplete evacuation with persistence of retained intrauterine products, increased risk of intrauterine adhesions, curettage extending beyond the basalis layer, and uterine perforation. In fact, at hysteroscopy, the incidence of intrauterine adhesions was found to be as high as 50% in patients who were subjected to repeat curettage after prior incomplete blind curettage [6]. These factors have augmented importance in subfertile patients in whom an intact and optimal endometrial milieu is ideal for successful implantation.

More recently, hysteroscopic removal has been proposed in the literature as being a superior option to blind curettage [4–7]. In their systematic review, Hooker et al. (2016) noted that, compared to blind curettage, hysteroscopic evacuation of RPOC was associated with fewer incomplete evacuations (29% vs. 1%), intrauterine adhesions (30% vs. 13%), and a trend towards earlier conception in the hysteroscopic group [8]. Resectoscopy, morcellation, or use of a hysteroscopic grasper may be used to achieve tissue removal [8]. Given the various options available for hysteroscopic uterine evacuation, the optimal hysteroscopic tissue removal device warrants consideration.

We present two (2) patients successfully managed at the Centre for Assisted Reproduction (CARE) Unit with RPOC using the Bigatti hysteroscopic tissue removal system after previous surgical treatment with blind curettage. Benefits of this approach included a short set up and procedure time and no intraoperative or postoperative complications.

## 2. Case

**Figure 1:**
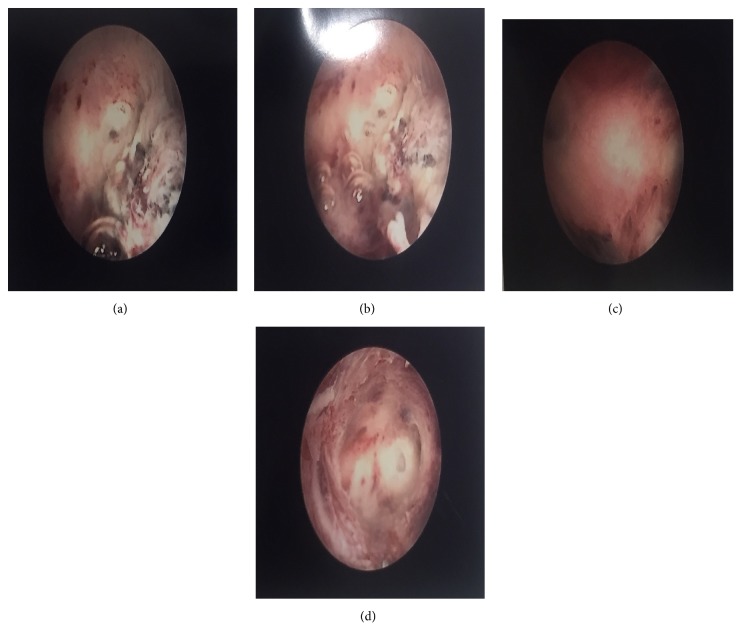
Retained products of conception: (a) and (b): prehysteroscopic morcellation and (c) and (d): postmorcellation appearance with the Intrauterine Bigatti Shaver system.

(1) A 30 y.o. P_0_^+1^ underwent a successful ovulation induction with an intrauterine conception but subsequently suffered a missed miscarriage. She initially underwent a suction curettage for the miscarriage but re-presented 3 months later with abnormal uterine bleeding: prolonged menstrual bleed and intermenstrual bleed. Ultrasound findings were suggestive of retained products on conception. She was offered and consented to hysteroscopic removal (under general anaesthesia) to minimize the risk of repeat retention. Intraoperatively, a 1.5 cm area of retained products of conception was seen close to the right ostium. Complete product removal was achieved during a 7-minute procedure with minimal blood loss (Figure 1).

(2) A 38 y.o. nulliparous female after in vitro fertilization and embryo transfer: The patient had a successful implantation but was subsequently diagnosed with a missed miscarriage. She had a spontaneous expulsion of products of conception and was scheduled for a repeat frozen embryo transfer. However, during ultrasound, she was noted to have retained products of conception and was offered hysteroscopic removal of the same. She had preoperative cervical ripening with misoprostol 400 mg per vaginum followed by hysteroscopic morcellation under general anaesthesia. Intraoperatively, a 1 cm area of product of conception was visualized at the posterior wall of the uterine cavity which was otherwise normal. The procedure was uncomplicated and lasted 6 minutes.

## 3. Discussion

The optimal management of RPOC necessitates complete evacuation of the retained products. This is important as failure to do so is associated with significant short and long term complications. Short term complications include incomplete evacuation and need for repeat procedure, infection, sepsis, haemorrhage, and uterine and cervical trauma [4, 9]. Long term complications include implantation failure due to a foreign body effect, abnormal placentation and formation of intrauterine adhesions and their potential adverse sequelae on reproductive outcomes in particular, miscarriages, and sub- or infertility [4, 7, 10]. Any method used to evacuate the uterus may be associated with the aforementioned complications. However, these risks have been found to be significantly greater in patients undergoing blind curettage [4, 7, 8, 11]. It should be noted that the majority of patients who present with retained products of conception are within the reproductive age group and may desire future fertility. As such, it is imperative that complete evacuation is achieved whilst simultaneously maintaining endomyometrial integrity [10]. Hysteroscopic evacuation increases this likelihood as it is done under direct vision [1, 9]. Consequently, there is an associated decrease in the need for repeat procedures, risk of uterine perforation, infection, endometrial trauma, and subsequent intrauterine adhesions and subfertility [3]. In their retrospective analysis, Ben Ami et al. (2014) noted that, compared to dilation and curettage (D&C), hysteroscopic removal of RPOC was associated with a shorter mean time to subsequent conception in addition to a lower rate of occurrence of newly diagnosed infertility problems [11]. Additional benefits include the identification and treatment of other uterine or endometrial pathology. Other surgical options include ultrasound guided curettage which is still a blind procedure and thus is associated with a greater likelihood of the previously mentioned risks [12].

Since its introduction in 1997, the expertise and use of hysteroscopy in the management of endometrial pathology have significantly increased. Pathology may be removed using a resectoscope loop, morcellation, or a hysteroscopic grasper [9, 12, 13]. Whereas resectoscopy is more commonly used, morcellation is less described in the management of retained products of conception. However, there is a risk of thermal spread and visceral injury with energy use during resectoscopy [14]. Such thermal spread may extend beyond the excision of the visible lesion to the basalis layer resulting in healing via repair instead of regeneration and thus an increased adhesiogenic risk [15]. Conversely, hysteroscopic morcellation does not require energy use thereby obviating the potential for visceral heat injury. In addition, compared to the resectoscope, the hysteroscopic morcellator utilizes a smaller hysteroscope thereby necessitating less cervical dilation [16]. Another significant benefit of the hysteroscopic morcellation technique is the associated clear operative view, which can be maintained due to concomitant suction of the tissue throughout, thereby reducing the number of scope reinsertions and unintended endometrial trauma [16]. The current available morcellators have an inbuilt suction apparatus that allows for immediate removal of resected tissue with no compromise to intracavitary visibility. The presence of floating tissue associated with resectoscopy often necessitates the use of a grasper to aid its removal thereby prolonging procedure time. This may also result in inadvertent uterine perforation and its attendant sequelae. Utilization of a morcellator is superior to the resectoscopy in this regard. These advantages may translate to the ability to perform hysteroscopic procedures previously confined to the operating room in an in-office setting with improvements in cost and efficiency.

Advantages of blind resection in the management of RPOC do exist. There is a shorter learning curve and preprocedure setup compared to operative hysteroscopy. It may be associated with lower costs in the short term as hysteroscopy requires the purchase of an additional resectoscope for each patient. Also, of significance is the avoidance of potential fluid overload associated with operative hysteroscopy.

In their meta-analysis of 392 patients with endometrial pathology, Li et al. concluded that hysteroscopic morcellation was associated with a higher operative success (odds ratio 4.5) rate and a shorter operative time among patients with endometrial lesions compared to resectoscopy [17]. With specific regard to the management of RPOC, hysteroscopic morcellation has only recently been described in the literature [18–20]. Findings from one study which randomized patients with RPOC to hysteroscopic morcellation versus hysteroscopic resection were as follows: whilst both approaches were safe with high rates of complete tissue removal, hysteroscopic morcellation was significantly faster than loop resection. Additionally, procedure time and number of scope reinsertions were also significantly lower in the morcellation group [20]. Similar findings were noted by van Dongen et al. (2008) [21]. In both cases we present, no adverse events were noted and the procedure was completed in less than ten minutes.

The Intrauterine Bigatti Shaver (IBS) was recently introduced as an option for hysteroscopic morcellation. It consists of a 6^0^ angled telescope with an integrated sheath and working channel into which a rigid shaver is inserted. The diameter of its outer sheath is 8 mm (24F) and an inner tube connected to a handpiece oscillates within the inner piece [22]. Descriptions in the literature regarding the use of morcellators include the TruClear and MyoSure Hysteroscopic Removal Systems [4, 7, 23, 24]. The use of this new system in the management of our two cases yielded results comparable to the limited cases of morcellation RPOC in the literature. In view of its benefits which include sustained intraoperative visibility, a short setup and procedure time, and definitive management of pathology, we advocate use of the IBS in the management of minimally symptomatic patients with focal retention of gestational products in whom complete uterine evacuation and simultaneous preservation of endometrial integrity are strongly desired. It is however ideal that prospective studies of larger populations be conducted to address this issue specifically addressing cost, comparison to other existing morcellating systems, potential unique complications, and the associated learning curve given its relatively recent introduction. Our pilot experience suggests that this might be the way forward in the future especially in the management of patients with prior incomplete curettage and reproductive desires.
